# Free amino acid–rich egg yolk protein hydrolysate promotes osteogenesis of MC3T3-E1 cells association with *β*-catenin nuclear translocation

**DOI:** 10.3389/fnut.2026.1774605

**Published:** 2026-02-19

**Authors:** Yulong Zheng, ChoYeon Park, HyeJi Hwang, Byung-Hak Kim, Sang Jae Park, Il-Jun Kang

**Affiliations:** 1 School of Public Health, North Sichuan Medical College, Nanchong, China; 2 Department of Food Science and Nutrition & the Korean Institute of Nutrition, Hallym University, Chuncheon, Republic of Korea; 3 Medience Co., Ltd., Chuncheon, Republic of Korea

**Keywords:** egg yolk protein hydrolysate, free amino acid, MC3T3-E1 osteoblasts, osteogenesis, *β*-catenin

## Abstract

**Background:**

Promoting osteogenesis is a key approach to preventing and improving bone metabolic diseases.

**Objective:**

This study investigated the impact of free amino acid-rich egg yolk protein hydrolysate (Y-PEP) on MC3T3-E1 osteoblast differentiation and mineralization.

**Methods:**

Free amino acids in Y-PEP were quantified using a high-speed amino acid analyzer. MC3T3-E1 cells were osteogenically induced with Y-PEP (5–100 μg/mL), and cell viability (72 h), alkaline phosphatase activity and collagen synthesis (day 9), as well as calcium deposition and osteocalcin production (day 18) were assessed. Moreover, runt-related transcription factor 2/osterix mRNA (qRT-PCR) and total/cytosolic/nuclear *β*-catenin and β-catenin phosphorylation (western blot) were measured.

**Results:**

Twenty-one amino acids, including leucine, lysine, arginine, glutamic acid, and valine, were identified and quantified in Y-PEP by comparing retention times and peak areas with amino acid mixture standard solutions. Y-PEP concentrations below 100 μg/mL had no impact on MC3T3-E1 osteoblast viability. Y-PEP enhanced osteoblast differentiation markers in a dose-dependent manner at concentrations from 5 to 100 μg/mL and promoted mineralization markers in mature osteoblasts dose-dependently at 25–50 μg/mL. The pro-osteogenic effect of Y-PEP may involve increasing total cellular *β*-catenin levels and promoting β-catenin nuclear translocation rate to upregulate transcription of osteogenesis-associated genes. The osteogenic activity of Y-PEP may result from the synergistic effects among signal transduction, metabolism, and mineral handling driven by its complex amino acid composition.

**Conclusion:**

Y-PEP can promote osteogenesis in MC3T3-E1 cells and has the potential to serve as a functional food ingredient for preventing or improving metabolic bone diseases.

## 1 Introduction

Bone tissue maintains homeostasis through osteoclast-mediated resorption and osteoblast-mediated formation (1). Fragility fractures occur when newly formed bone cannot adequately replace tissue removed during skeletal turnover due to intrinsic factors (including hormones, genetics, or aging) and extrinsic factors (including nutrition, inflammation, or disease) (2). In particular, decreased osteoblast activity is a central pathological feature of osteoporosis and other metabolic bone disorders (3, 4). Therefore, strategies to stimulate osteoblast differentiation and mineralization have become key approaches to supporting bone health and potentially preventing bone diseases (5–7).

Osteoblastogenesis is coordinated by several intersecting signaling pathways, among which the Wnt/*β*-catenin pathway plays a dominant role in osteogenesis (8, 9). The destruction complex typically ubiquitinates *β*-catenin to regulate its intracellular concentrations (10). Upon activation of Wnt signaling, *β*-catenin escapes N-terminal phosphorylation-mediated degradation, and cytoplasmic accumulation occurs (11). Stable β-catenin is transported into the nucleus and binds to transcription factors, inducing the expression of osteogenic genes, including runt-related transcription factor 2 (Runx2), osterix (Osx), and alkaline phosphatase/tissue-nonspecific isozyme (Alpl) (12). Therefore, *β*-catenin phosphorylation and its migration to the nucleus are proposed as potential pathways for osteogenic factor action. There is abundant evidence that food-derived peptides (13, 14) and free amino acids such as arginine (15) and glutamine (16) stimulate the Wnt/*β*-catenin signaling cascade.

Egg yolk protein is a by-product of yolk lecithin extraction, which can be produced in large quantities but has not been fully utilized (17, 18). Various functional peptides and free amino acids produced after hydrolysis of egg yolk protein have antioxidant, anti-inflammatory, and metabolic regulatory effects while ensuring safety for long-term consumption (19–21). Moreover, egg yolk protein hydrolysate (Y-PEP) also exhibits strong metal ion chelation capacity that promotes the absorption of minerals such as calcium and iron (22, 23). These bioactivities suggest that Y-PEP has the potential to indirectly or directly affect osteogenesis but have not yet been scientifically confirmed.

This study investigated the impact of Y-PEP on the differentiation and mineralization of MC3T3-E1 osteoblasts. Specifically, 3 objectives are anticipated: (i) characterize the free amino acid profile of Y-PEP; (ii) evaluate its effects on early differentiation and late mineralization; and (iii) examine whether these effects are associated with increased *β*-catenin stability, nuclear translocation, and upregulation of downstream osteogenic gene expression. This study will provide preliminary evidence supporting the potential role of food-derived amino acid nutritional supplements in bone health.

## 2 Methods and materials

### 2.1 Preparation of Y-PEP

Chicken egg yolk protein was purchased from Solus Biotech Co., Ltd. (Iksan, Korea). Y-PEP was prepared by sequential enzymatic hydrolysis with a 2.5–5% enzyme/substrate ratio under pH 7.0 and 58 °C for 4–6 h. After hydrolysis, the enzyme was inactivated at 85 °C for 1 h and then centrifuged at 3,000 rpm for 20 min. The supernatant was passed through a 1 μm filter (Sigma-Aldrich, St Louis, MO, USA) before being concentrated and dried to produce Y-PEP.

### 2.2 Analysis of free amino acids in Y-PEP

The amino acids in Y-PEP were quantitatively analyzed using a high-speed amino acid analyzer (L-8900, Hitachi High-Technologies Corporation, Tokyo, Japan) equipped with an ion-exchange packed column (4.6 × 60 mm, Hitachi High-Technologies Corporation), and post-column derivatization was carried out using a ninhydrin coloring solution kit (Wako Pure Chemical Corporation, Osaka, Japan). Gradient elution and calibration were performed using the Kanto physiological fluid (PF) buffer system (PF-1–PF-4 and regeneration buffer, Kanto Chemical, Tokyo, Japan) and Type B/Type AN-II amino acid mixture standard solution (AAMS; Wako Pure Chemical Corporation). The flow rate of pump 1 (PF buffer system) was set at 0.35 mL/min, while that of pump 2 (ninhydrin coloring solution) was set at 0.30 mL/min. Y-PEP and AAMS were analyzed by comparison with the ninhydrin reaction assay to determine their dilution concentrations prior to injection (20 μL) (24). Detection was performed at 570 nm for primary amino acids and at 440 nm for secondary amino acids. Amino acids were identified by retention time comparison with AAMS and converted to concentrations by calculating peak areas using the calibration curve generated with EZ Chrom Elite software (version 3.1.5b, Scientific Software, Pleasanton, CA, USA). Gradient elution conditions and reaction temperatures are provided in Supplementary Table S1.

### 2.3 Cell culture and differentiation

MC3T3-E1 mouse osteoblasts were purchased from the American Type Culture Collection (Manassas, VA, USA) and cultured at 37 °C and 5% CO₂ in *α*-minimum essential medium (α-MEM; Gibco-Invitrogen, Grand Island, NY, USA) supplemented with 10% fetal bovine serum (Cytiva HyClone, Logan, UT, USA) and 1% penicillin/streptomycin (WelGene, Gyeongsan, South Korea). When the cells reached approximately 90% confluence, the medium was replaced with *α*-MEM containing 10 mM *β*-glycerophosphate and 50 μg/mL ascorbic acid (differentiation medium; Sigma-Aldrich) to induce osteogenesis. The osteoblast differentiation medium was changed every 3 days according to the previous studies (25, 26).

### 2.4 Cell viability assay

MC3T3-E1 osteoblasts were seeded at a density of 1 × 10^4^ cells/well in 96-well plates. After 24 h, the medium containing Y-PEP (5–200 μg/mL) was replaced and cultured for 72 h. Cell viability was measured at 450 nm after adding 10 μL of the Cellrix viability assay kit (MediFab, Seoul, Korea) and incubating at 37 °C for 2 h.

### 2.5 ALP (alkaline phosphatase) activity and collagen production assay

MC3T3-E1 osteoblasts were seeded at a density of 1 × 10^4^ cells/well in 96-well plates or 5 × 10^4^ cells/well in 24-well plates. After 24 h, differentiation was induced with differentiation medium containing 5–100 μg/mL Y-PEP or 100 nM 17-*β*-estradiol (E2; Sigma-Aldrich) for 8 days. On day 9, ALP activity and collagen production in differentiated cells were measured using the TRACP & ALP assay kit (Takara Bio, Japan) and Sirius red collagen detection kit (Chondrex, Woodinville, WA, USA), respectively, both according to the provided instructions.

### 2.6 Calcium deposition and osteocalcin assay

MC3T3-E1 osteoblasts were seeded at a density of 5 × 10^4^ cells/well in 24-well plates. After 24 h, differentiation was induced with differentiation medium containing 5–100 μg/mL Y-PEP or 100 nM E2. Calcium deposition and osteocalcin levels were determined after an 18-day culture using the osteogenesis assay kit (Merck Millipore, Burlington, MA, USA) and the osteocalcin ELISA kit (Takara Bio), respectively, both according to the provided instructions.

### 2.7 Osteogenic gene expression analysis

MC3T3-E1 osteoblasts were cultured in differentiation medium containing 5–100 μg/mL Y-PEP or 100 nM E2 for 3 days, and total RNA was extracted using the RNeasy plus mini kit (Qiagen, Germantown, MD, USA). Quantify RNA with a micro-volume spectrophotometer (BioSpec-nano, Kyoto, Japan) and confirm the OD260/280 value exceeds 1.8. For quantitative real-time PCR analysis, 2 μg of total RNA was reverse transcribed into cDNA using the HyperScript RT master mix kit (GeneAll, Seoul, Korea) and amplified in a reaction mixture containing the Rotor-Gene™ SYBR green kit (Qiagen) with the Rotor-Gene 3,000 PCR system (Corbett Research, Sydney, Australia). The primer sets are shown in Table 1.

**Table 1:** The primer sets for quantitative real-time PCR.

Gene	Sequences	Genebank no.
Runx2	F 5’-AGGGACTATGGCGTCAAACA-3′	XM_029471398.1
	R 5’-GGCTCACGTCGCTCATCTT-3′	
Osx	F 5’-CGCTTTGTGCCTTTGAAAT-3′	XM_006520519.4
	R 5’-CCGTCAACGACGTTATGC-3′	
Gapdh	F 5’-TGGGTGTGAACCATGAGAAG-3′	XM_029478683.1
	R 5’-GCTAAGCAGTTGGTGGTGC-3′	

Runx2: runt-related transcription factor 2, Osx: osterix, Gapdh: glyceraldehyde-3-phosphate dehydrogenase.

### 2.8 *β*-Catenin expression analysis

MC3T3-E1 osteoblasts were cultured in differentiation medium containing 10–100 μg/mL Y-PEP for 3 days, and total protein was extracted using HEPES lysis buffer with Triton X-100 (Thermo Scientific, Waltham, MA, USA). Cytoplasmic and nuclear proteins were isolated from total cell lysates using the Nuclear/Cytosol fractionation kit (BioVision, Milpitas, CA, USA). Protein was quantified using a BCA protein assay kit (Thermo Scientific), and 50 μg of protein were separated by 10% SDS-PAGE and transferred to the PVDF membrane. The membrane was incubated in TBST with 5% skim milk for 1 h and then incubated with primary antibodies overnight at 4 °C. After incubation with the appropriate secondary antibody for 1 h, the protein bands were visualized using the immobilon western chemiluminescent HRP substrate (Merck Millipore) and subsequently quantified with the ImageQuant LAS 500 imaging system (GE Healthcare Bio-Sciences AB, Uppsala, Sweden). *β*-catenin, phospho-β-catenin (p-β-catenin), and β-actin antibodies were purchased from Cell Signaling Technology (Danvers, MA, USA).

### 2.9 Statistical analysis

All the data were represented as the mean ± standard error of the mean (SEM). Statistical analysis was performed with SPSS software 25.0 (IBM Corp., Armonk, NY, USA). Significance was determined by one-way analysis of variance (ANOVA) followed by Tukey’s *post hoc* test, and *p* < 0.05 was considered statistically significant.

## 3 Results

### 3.1 Y-PEP contains a variety of free amino acids

**Figure 1:**
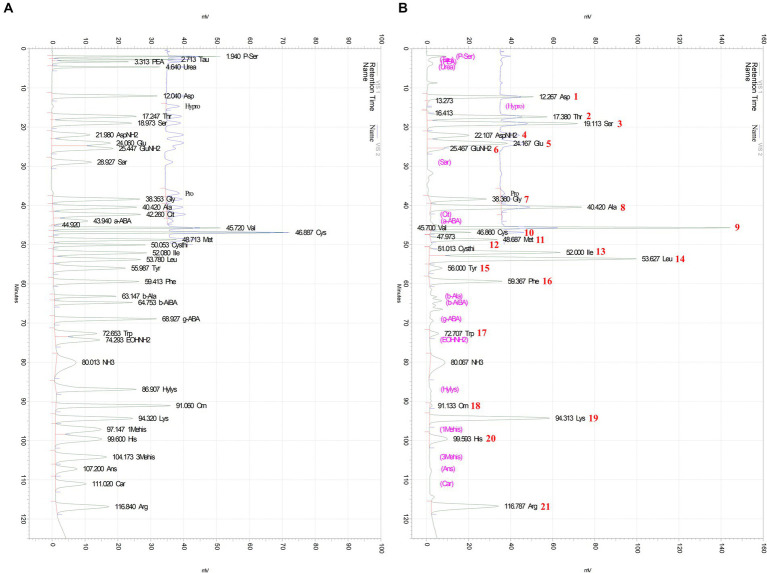
Amino acid separation chromatograms of AAMS and Y-PEP. **(A)** Separation chromatogram of Type B/Type AN-II amino acid mixture standard solution (AAMS) and **(B)** Separation chromatogram of egg yolk protein hydrolysate (Y-PEP). Amino acids were detected at 440 and 570 nm, respectively, and identified and quantified by comparing the retention times of the AAMS and Y-PEP chromatograms. Peak numbers shown in the chromatogram correspond to the amino acids listed in Table 2.

As shown in Figure 1, amino acids in Y-PEP were identified and quantified by matching their retention times with AAMS. A total of 21 amino acids or aminated compounds were identified and quantified, and the remaining compounds were below the detection limit. To calculate the composition, the instrument detected non–amino-acid species (ammonia and urea) but did not include them in the composition.

Y-PEP is mainly composed of branched-chain amino acids, basic residues, and acidic residues including amide forms. The 5 amino acids with the highest contents were leucine (11.89%), lysine (8.43%), arginine (8.10%), glutamic acid (7.99%), and valine (7.79%), whereas non-proteinogenic amino acids such as ornithine (0.09%) and cystathionine (0.05%) were present in lower amounts (Table 2).

**Table 2:** Amino acid composition of Y-PEP.

Peak number	Amino acid	Retention time (min)	Concentration (μmol/L)	Content (%)
1	Aspartic acid	12.267	202066.081	4.98
2	Threonine	17.380	282773.759	6.23
3	Serine	19.113	373289.543	7.26
4	Asparagine	22.107	215059.228	5.26
5	Glutamic acid	24.167	293638.873	7.99
6	Glutamine	25.467	34724.051	0.94
7	Glycine	38.360	128645.186	1.79
8	Alanine	40.420	364633.399	6.01
9	Valine	45.700	359632.517	7.79
10	Cysteine	46.860	34000.078	1.51
11	Methionine	48.687	108944.916	3.01
12	Cystathionine	51.013	1299.790	0.05
13	Isoleucine	52.000	290830.958	7.06
14	Leucine	53.627	489926.444	11.89
15	Tyrosine	56.000	36448.002	1.22
16	Phenylalanine	59.367	175884.396	5.38
17	Tryptophan	72.707	41938.315	1.59
18	Ornithine	91.133	3656.226	0.09
19	Lysine	94.313	311560.822	8.43
20	Histidine	99.593	76968.575	2.21
21	Arginine	116.787	251408.518	8.10

The amino acid content percentage in egg yolk protein hydrolysate (Y-PEP) was normalized based on mass concentration.

### 3.2 Y-PEP treatment concentration range was selected based on cell viability

**Figure 2:**
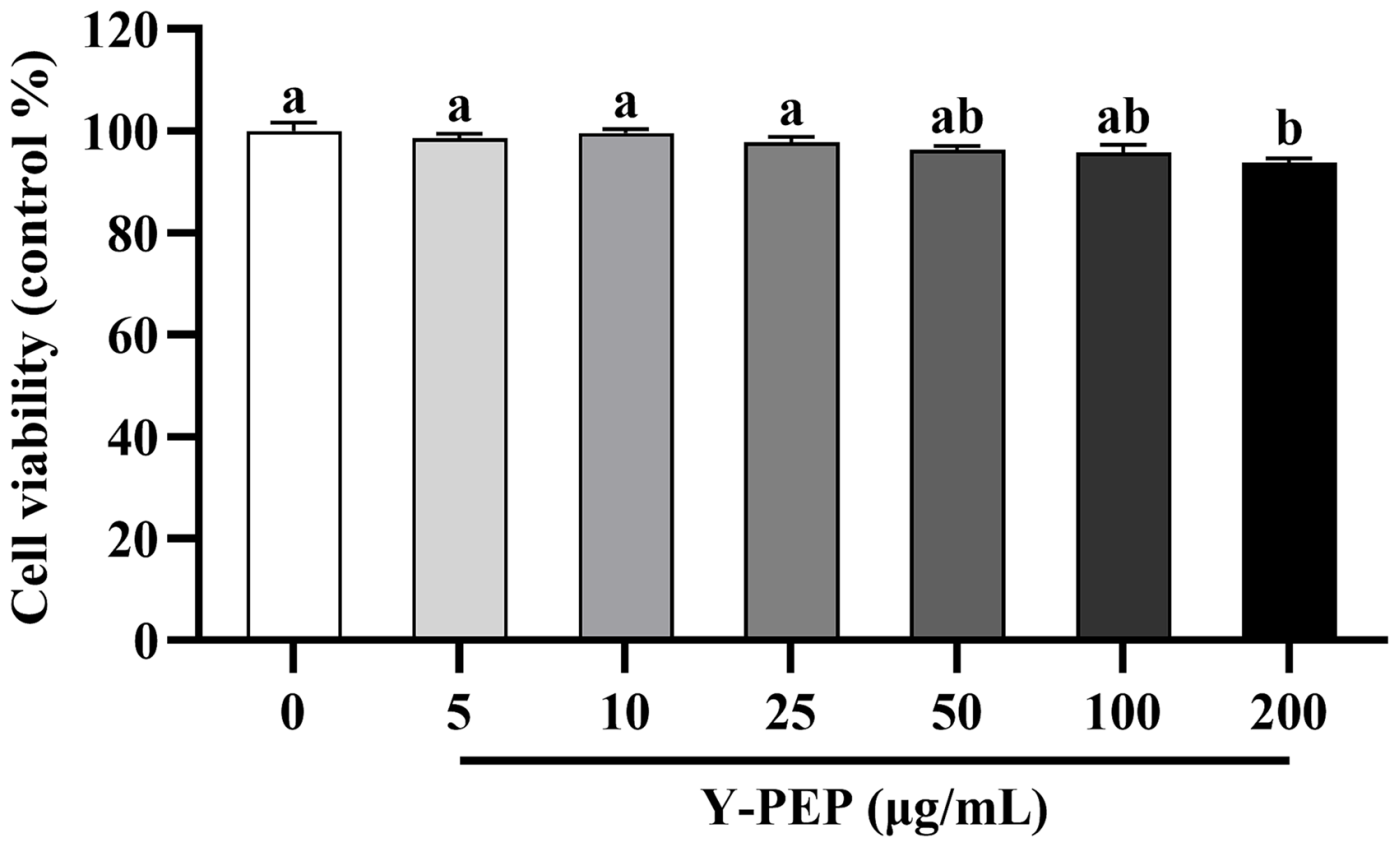
Effect of Y-PEP on cell viability of MC3T3-E1 osteoblasts. Cell viability was measured after treating MC3T3-E1 osteoblasts with 5–200 μg/mL egg yolk protein hydrolysate (Y-PEP) for 72 h. The control group received without Y-PEP treatment (0 μg/mL). Values are expressed as mean ± SEM (*n* = 3), and significant differences were determined by one-way analysis of variance (ANOVA) followed by Tukey’s *post hoc* test. Different lowercase letters denote statistically differences at *p* < 0.05.

As shown in Figure 2, Y-PEP levels below 100 μg/mL did not affect the cell viability of MC3T3-E1 osteoblasts. Compared with the control group (0 μg/mL), cell viability was significantly reduced at a Y-PEP concentration of 200 μg/mL. Therefore, the treatment concentration range of Y-PEP in the differentiation induction experiment was 5–100 μg/mL.

### 3.3 Y-PEP promoted the differentiation of MC3T3-E1 osteoblasts

**Figure 3:**
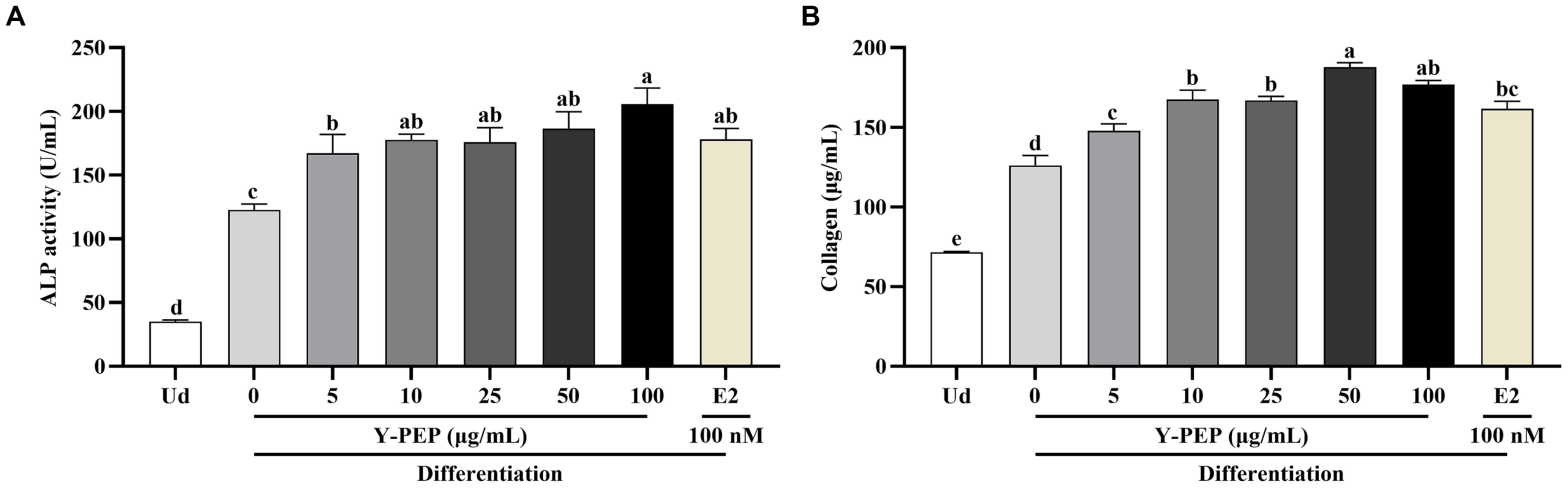
Effects of Y-PEP on the differentiation of MC3T3-E1 osteoblasts. **(A)** Effect of 5–100 μg/mL egg yolk protein hydrolysate (Y-PEP) on alkaline phosphatase (ALP) activity in MC3T3-E1 osteoblasts differentiated for 9 days. **(B)** Effect of 5–100 μg/mL Y-PEP on collagen synthesis in MC3T3-E1 osteoblasts differentiated for 9 days. Ud: undifferentiated group, Y-PEP 0 μg/mL: differentiated control group, E2: the group differentiated and treated with 100 nM 17-β-estradiol. Values are expressed as mean ± SEM (*n* = 3), and significant differences were determined by one-way analysis of variance (ANOVA) followed by Tukey’s post hoc test. Different lowercase letters denote statistically differences at *p* < 0.05.

As shown in Figure 3, the ALP activity and collagen production in the differentiated control group (0 μg/mL) were significantly higher than those in the undifferentiated group (Ud). Y-PEP significantly enhanced ALP activity in MC3T3-E1 osteoblasts in a dose-dependent manner within the 5–100 μg/mL concentration range (Figure 3A). When Y-PEP concentrations exceeded 10 μg/mL, its ALP activity-enhancing effect was comparable to that of E2, and exerted a stronger impact than E2 at 100 μg/mL.

Similarly, Y-PEP in the range of 5–50 μg/mL dose-dependently promoted collagen synthesis in MC3T3-E1 osteoblasts (Figure 3B). Compared with the differentiated control group, Y-PEP at concentrations above 10 μg/mL promoted collagen synthesis more effectively than E2.

### 3.4 Y-PEP enhanced mineralization in differentiated MC3T3-E1 osteoblasts

**Figure 4:**
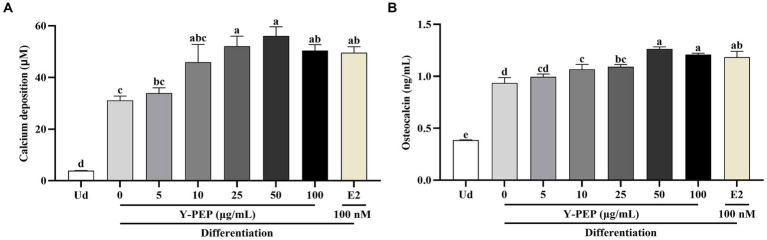
Effects of Y-PEP on mineralization of differentiated MC3T3-E1 osteoblasts. Effects of 5–100 μg/mL egg yolk protein hydrolysate (Y-PEP) on **(A)** calcium deposition and **(B)** osteocalcin synthesis in MC3T3-E1 osteoblasts differentiated for 18 days. Ud: undifferentiated group, Y-PEP 0 μg/mL: differentiated control group, E2: the group differentiated and treated with 100 nM 17-β-estradiol. Values are expressed as mean ± SEM (*n* = 3), and significant differences were determined by one-way analysis of variance (ANOVA) followed by Tukey’s post hoc test. Different lowercase letters denote statistically differences at *p* < 0.05.

As shown in Figures 4A,B, calcium deposition and osteocalcin production in the differentiated control group were significantly higher than those in the undifferentiated group. Y-PEP at concentrations above 25 μg/mL significantly promoted calcium deposition and stimulated osteocalcin synthesis in the differentiated control group. The calcium deposition and osteocalcin synthesis levels in the 50 μg/mL Y-PEP-treated group exhibited the highest level, which was significantly higher than those in the E2-treated group. In contrast, the treatment concentration of Y-PEP exhibited a downward trend starting from 100 μg/mL.

### 3.5 Y-PEP upregulated the expression of osteogenic genes

**Figure 5:**
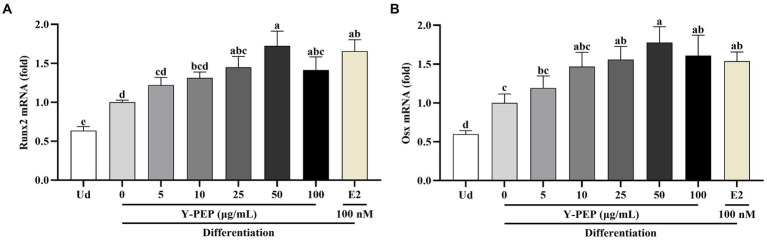
Effects of Y-PEP on osteogenic gene expression in MC3T3-E1 osteoblasts. Effects of 5–100 μg/mL egg yolk protein hydrolysate (Y-PEP) on **(A)** runt-related transcription factor 2 (Runx2) and **(B)** osterix (Osx) gene expression levels in MC3T3-E1 osteoblasts differentiated for 3 days. Ud: undifferentiated group, Y-PEP 0 μg/mL: differentiated control group, E2: the group differentiated and treated with 100 nM 17-β-estradiol. The gene values were normalized to the glyceraldehyde-3-phosphate dehydrogenase (Gapdh) gene. Values are expressed as mean ± SEM (*n* = 3), and significant differences were determined by one-way analysis of variance (ANOVA) followed by Tukey’s post hoc test. Different lowercase letters denote statistically differences at *p* < 0.05.

As shown in Figures 5A,B, Runx2 and Osx gene levels in the differentiated control group were significantly higher than in the undifferentiated group. Y-PEP treatment upregulated the expression levels of Runx2 and Osx genes in the differentiated control group, with significance at 25 μg/mL and a maximum at 50 μg/mL. In the Y-PEP-treated group, Runx2 and Osx gene levels instead began to decline when the concentration exceeded 50 μg/mL. Therefore, the Y-PEP treatment concentration in subsequent *β*-catenin protein expression experiments was 10–100 μg/mL.

### 3.6 Y-PEP increased the nuclear translocation rate of *β*-catenin

**Figure 6:**
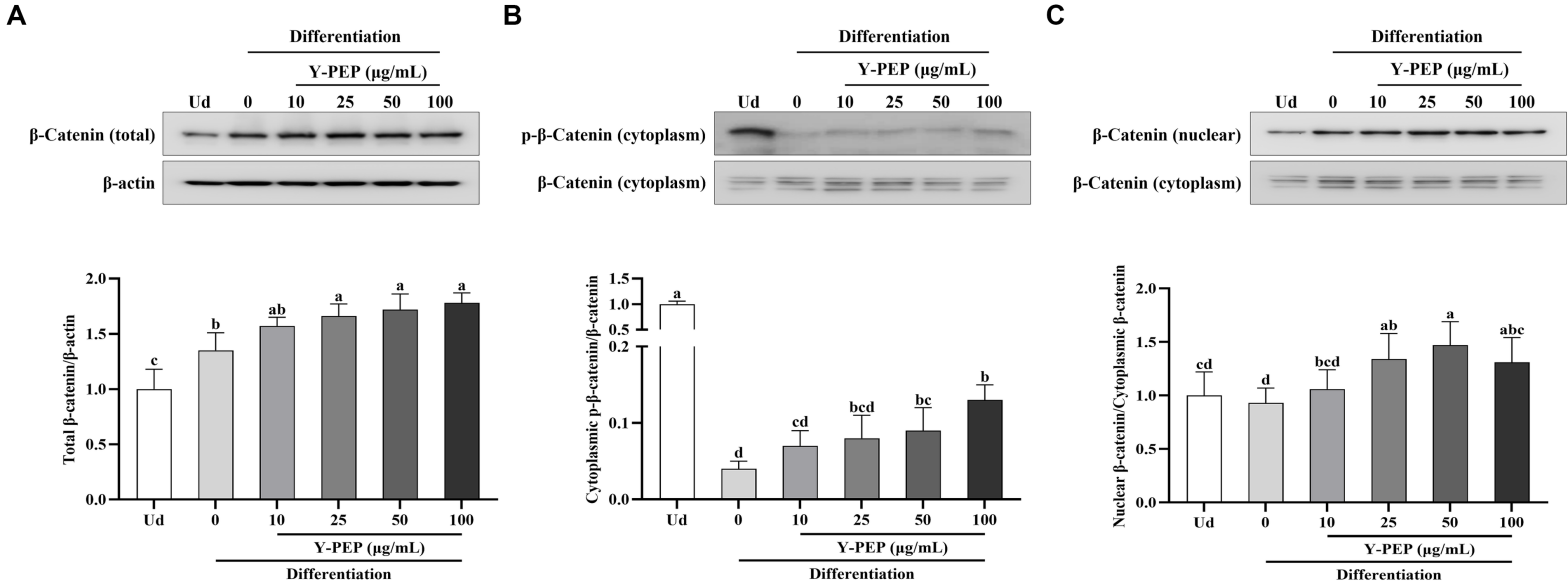
Effect of Y-PEP on β-catenin protein expression in MC3T3-E1 osteoblasts. Effects of 10–100 μg/mL egg yolk protein hydrolysate (Y-PEP) on **(A)** total β-catenin protein expression, **(B)** cytoplasmic β-catenin phosphorylation levels, and **(C)** β-catenin nuclear translocation rate in MC3T3-E1 osteoblasts differentiated for 3 days. Ud: undifferentiated group, Y-PEP 0 μg/mL: differentiated control group. Values are expressed as mean ± SEM (*n* = 3), and significant differences were determined by one-way analysis of variance (ANOVA) followed by Tukey’s post hoc test. Different lowercase letters denote statistically differences at *p* < 0.05.

The total β-catenin protein level in the differentiated control group was significantly higher than that in the undifferentiated group, and Y-PEP above 25 μg/mL dose-dependently increased *β*-catenin protein expression in the differentiated control group (Figure 6A). The cytoplasmic p-*β*-catenin in the differentiated control group was significantly decreased compared with that in the undifferentiated group. Y-PEP treatment increased *β*-catenin phosphorylation level in the differentiated control group, but the effect was significant only at concentrations above 50 μg/mL (Figure 6B). The β-catenin nuclear translocation rate in the differentiated control group did not differ from that in the undifferentiated group. Y-PEP dose-dependently increased the β-catenin nuclear translocation rate in the differentiated control group and was significant at concentrations above 25 μg/mL (Figure 6C). However, the nuclear translocation rate of β-catenin began to decrease in the 100 μg/mL Y-PEP-treated group.

## 4 Discussion

The present study revealed that Y-PEP functions as an amino acid–rich nutritional source to promote osteogenesis in MC3T3-E1 cells. The currently observed endpoint indicators align with the established osteoblast differentiation trajectory. Early-stage directed differentiation is characterized by increased ALP activity and collagen synthesis, while late-stage maturation is marked by enhanced calcium deposition and osteocalcin secretion, along with the activation of osteogenic transcription programs (27). Runx2 and Osx are widely recognized as key transcription factors that drive osteoblast differentiation and maturation. Their synergistic upregulation and the improvement of osteogenic function indicators support the anabolic phenotype of osteoblasts in this study model (28). Concurrently, the observed increase in *β*-catenin abundance and nuclear accumulation aligns with the potential involvement of the Wnt/β-catenin signaling in these osteogenic processes (12). These findings suggest that Y-PEP is a multicomponent osteogenic stimulator that involves at least the classical Wnt/β-catenin signaling pathway, thereby driving bone matrix production.

Y-PEP is rich in branched-chain amino acids (valine and leucine), cationic residues (lysine and arginine), and cysteine/glutamine. Among these amino acids, glutamine is required for Wnt-driven metabolism (29), arginine regulates Wnt signaling (15), and cysteine activates the Wnt/β-catenin pathway by supplying exogenous H₂S (30). Furthermore, leucine and arginine interact with Wnt signaling as mammalian target of rapamycin (mTOR)-dependent anabolic modulators (31). In addition to signal transduction, cationic residues and acidic side chains (aspartic acid and glutamic acid) can serve as calcium-binding motifs (32, 33), while the peptide-calcium complex can induce protein-mineral co-deposition (32, 34). These complementary mechanisms provide a coherent framework for the osteogenesis-promoting effects of Y-PEP. More broadly, the findings of this study are consistent with a growing body of evidence that food-derived peptides or hydrolysates can enhance osteoblast activity and osteogenesis, supporting the notion that nutrient-derived bioactive substances can be used to promote bone formation (35). Notably, Y-PEP matches or surpasses E2 in promoting effects on several osteogenic markers. E2 was used as a reference osteogenic stimulant (36), but may cause inherent side effects associated with hormone therapy (37). Y-PEP is a food-derived preparation that may support osteogenesis through a non-estrogen mechanism. This distinction is crucial for populations concerned about hormonal safety.

The notable characteristic of Y-PEP is that its pro-osteogenic effects and *β*-catenin nuclear shuttling occur within a narrow dose range and are accompanied by hormetic behavior. This phenomenon was common in bioactive peptide mixtures (38–40), suggesting that Y-PEP may be involved in both pro-osteogenic and counter-regulatory processes. The decline in osteogenesis of Y-PEP at 100 μg/mL was not caused by obvious cytotoxicity but rather by pathway-level feedback. The moderate increase in cytoplasmic *β*-catenin phosphorylation at 100 μg/mL of Y-PEP may explain the reduction in osteogenesis. This is consistent with the dose-dependent upregulation of Runx2/Osx transcription and the subsequent increase of downstream ALP activity, collagen, and mineral accumulation observed within the 25–50 μg/mL concentration range of Y-PEP. Other possible mechanisms also include amino acid load stress responses to attenuate Wnt/β-catenin signaling (41–43) or calcium-binding molecules that excessively chelate free Ca^2+^ to limit mineralization (44–46). The *in vitro* effective concentration of Y-PEP (25–50 μg/mL) should be regarded as a validation of efficacy rather than a physiological dose. Oral hydrolysates are further broken down into free amino acids and small peptides in the gastrointestinal tract, and systemic exposure depends on absorption and metabolism (47). Furthermore, food-derived peptides have been detected in human circulation, their bioavailability remains plausible (48). Therefore, this evidence supports the relevance of Y-PEP as a food-derived nutrient in the development of functional foods or nutritional supplements. However, elucidating the dose-exposure relationship and identifying key active ingredients requires precise fractionation of Y-PEP for targeted analysis, which presents a formidable challenge.

Despite the above findings, several limitations should be noted. First, the existing data support involvement of the *β*-catenin signaling pathway, but no pathway perturbation experiments have been conducted. Therefore, the possibility that β-catenin activation occurs in parallel with other pathways cannot be ruled out. Secondly, this study quantitatively analyzed the free amino acids in Y-PEP and described it as a free amino acid-rich proteolytic hydrolysate. However, it should be noted that egg yolk protein hydrolysates are typically mixtures of free amino acids and small peptides, both of which may contribute to osteogenic bioactivity (49). Therefore, the current results should be interpreted as reflecting the activity of a multicomponent mixture, in which free amino acids and peptides may have additive or synergistic effects. Future research in these areas will help to more deeply elucidate the translational significance of Y-PEP in promoting bone health.

## 5 Conclusion

From a sustainability and practicality perspective, Y-PEP is derived from food-grade egg protein and can be produced on a large scale through enzymatic hydrolysis, thereby enabling the value-added utilization of egg by-product resources and supporting sustainable upcycling within the food chain. In conclusion, the complex amino acid composition of Y-PEP aligns with its multi-targeted mode of action, which encompasses signal transduction, metabolism, and mineral-related processes. These findings provide a theoretical basis for further exploration of Y-PEP as a food-derived nutritional to support bone anabolism, while acknowledging the need for additional mechanistic and *in vivo* studies.

## Data Availability

The original contributions presented in the study are included in the article/Supplementary material, further inquiries can be directed to the corresponding author.
