# Supplementary material for: Free amino acid–rich egg yolk protein hydrolysate promotes osteogenesis of MC3T3-E1 cells association with β-catenin nuclear translocation
Source: Front Nutr. 2026 Feb 19;13:1774605. doi: 10.3389/fnut.2026.1774605 (PMC12960115; doi:10.3389/fnut.2026.1774605)
Supplement: Supplementary file 1 [file Table_1.docx]

Table S1. Gradient elution conditions and reaction temperature

| Time  (min) | %B1 | %B2 | %B3 | %B4 | %B5 | Temperature  (°C) | %R1 | %R2 | %R3 |
| --- | --- | --- | --- | --- | --- | --- | --- | --- | --- |
| 0.0 | 100 | 0 | 0 | 0 | 0 | 38 | 50 | 50 | 0 |
| 22.9 | 100 | 0 | 0 | 0 | 0 | 30 | 50 | 50 | 0 |
| 23.0 | 80 | 20 | 0 | 0 | 0 | 60 | 50 | 50 | 0 |
| 35.6 | 70 | 30 | 0 | 0 | 0 | 60 | 50 | 50 | 0 |
| 35.7 | 10 | 90 | 0 | 0 | 0 | 60 | 50 | 50 | 0 |
| 38.8 | 10 | 90 | 0 | 0 | 0 | 40 | 50 | 50 | 0 |
| 46.3 | 0 | 100 | 0 | 0 | 0 | 40 | 50 | 50 | 0 |
| 53.7 | 0 | 100 | 0 | 0 | 0 | 70 | 50 | 50 | 0 |
| 53.8 | 0 | 0 | 100 | 0 | 0 | 70 | 50 | 50 | 0 |
| 72.7 | 0 | 0 | 100 | 0 | 0 | 45 | 50 | 50 | 0 |
| 74.0 | 60 | 0 | 0 | 40 | 0 | 45 | 50 | 50 | 0 |
| 79.8 | 0 | 0 | 0 | 100 | 0 | 45 | 50 | 50 | 0 |
| 87.3 | 0 | 20 | 0 | 80 | 0 | 45 | 50 | 50 | 0 |
| 98.3 | 0 | 20 | 0 | 80 | 0 | 70 | 50 | 50 | 0 |
| 105.9 | 0 | 0 | 0 | 100 | 0 | 70 | 50 | 50 | 0 |
| 119.7 | 0 | 0 | 0 | 0 | 100 | 70 | 50 | 50 | 0 |
| 123.4 | 0 | 0 | 0 | 0 | 100 | 70 | 0 | 0 | 100 |
| 129.3 | 100 | 0 | 0 | 0 | 0 | 70 | 0 | 0 | 100 |
| 132.9 | 100 | 0 | 0 | 0 | 0 | 38 | 0 | 0 | 100 |
| 134.0 | 100 | 0 | 0 | 0 | 0 | 38 | 50 | 50 | 0 |
| 157.3 | 100 | 0 | 0 | 0 | 0 | 38 | 50 | 50 | 0 |

B: Kanto physiological fluid (PF) buffer system (mixing pump 1), B1: PF-1, B2: PF-2, B3: PF-3, B4: PF-4, B5: regeneration buffer, R: Wako ninhydrin coloring solution kit (mixing pump 2), R1: ninhydrin solution, R2: ninhydrin buffer, R3: 5% ethanol. The flow rate of mixing pump 1 was set at 0.35 mL/min, while that of mixing pump 2 was set at 0.30 mL/min.
